# Effect of Individual Agents on Time to Culture Conversion in *Mycobacterium avium* Complex Pulmonary Disease

**DOI:** 10.1093/ofid/ofaf138

**Published:** 2025-03-07

**Authors:** Joong-Yub Kim, Yunhee Choi, Jae-Joon Yim, Nakwon Kwak

**Affiliations:** Biomedical Research Institute, Seoul National University Hospital, Seoul, Republic of Korea; Department of Microbiology, Harvard Medical School, Boston, Massachusetts, USA; Division of Medical Statistics, Medical Research Collaborating Center, Seoul National University Hospital, Seoul, Republic of Korea; Division of Pulmonary and Critical Care Medicine, Department of Internal Medicine, Seoul National University Hospital, Seoul, Republic of Korea; Department of Internal Medicine, Seoul National University College of Medicine, Seoul, Republic of Korea; Division of Pulmonary and Critical Care Medicine, Department of Internal Medicine, Seoul National University Hospital, Seoul, Republic of Korea; Department of Internal Medicine, Seoul National University College of Medicine, Seoul, Republic of Korea

**Keywords:** antibiotic, nontuberculous mycobacteria, respiratory infection, rifampicin, time-varying

## Abstract

In a cohort of 534 patients treated for *Mycobacterium avium* complex pulmonary disease, those who failed to achieve culture conversion were older, had higher proportions of males and cavity presence, were more likely to receive clofazimine and aminoglycosides, but less likely to receive rifampicin, and had a shorter overall treatment duration. Time-varying analysis of individual drug effects on time to culture conversion identified rifampicin as being associated with a reduced culture conversion rate (adjusted hazard ratio, 0.959; 95% confidence interval, .924–.995; *P* = .027), suggesting a potentially negative effect on *Mycobacterium avium* complex pulmonary disease outcomes, whereas other drugs showed no significant association.

*Mycobacterium avium* complex (MAC) is the leading cause of nontuberculous mycobacterial (NTM) infections, resulting in pulmonary disease (PD) that necessitates prolonged macrolide-based multidrug therapy [1]. However, the effects of individual drugs remain uncertain owing to reliance on observational studies and the lack of randomized clinical trials [1]. During prolonged treatment, approximately one third of patients require regimen changes owing to persistent culture positivity, adverse events, or drug interactions [2], complicating the assessment of individual drug effectiveness. Consequently, individual antibiotic duration varies among patients, potentially introducing bias in the assessment of each drug's true impact; however, most studies fail to account for this variation [3]. In this study, we aimed to evaluate the effect of commonly used drugs for MAC-PD on treatment outcomes, specifically time to negative culture conversion, while adjusting for individual antibiotic duration using detailed longitudinal data.

## METHODS

This study included patients aged ≥19 years who met the diagnostic criteria for MAC-PD, characterized by pulmonary or systemic symptoms and radiologic findings consistent with NTM-PD after excluding alternative diagnoses, along with microbiological confirmation through at least 2 positive NTM cultures from separate expectorated sputum or at least 1 positive culture from a bronchoscopic or histologic specimen [1]. MAC species were identified using molecular methods [4]. All patients received treatment at Seoul National University Hospital between 1 January 2009 and 31 December 2021. The analysis focused on patients treated with macrolide/ethambutol-based regimens, with rifampicin, clofazimine, and parenteral aminoglycosides (amikacin, streptomycin) administered at the attending physician's discretion. In most cases, aminoglycosides were recommended for patients with severe disease at baseline, as assessed by the severity of pulmonary and systemic symptoms, as well as the extent of bronchiectasis and cavitary lesions [5], or for those who failed to achieve culture conversion and experienced disease progression despite more than 6 months of treatment. Patients were followed up every 1–3 months after treatment initiation. At each visit, sputum samples were collected for acid-fast bacilli smears and mycobacterial cultures, and chest radiographs were performed. Baseline data at treatment initiation included age, sex, body mass index, the presence of cavitary lesions on chest computed tomography scans, macrolide resistance [6, 7], and immunosuppressive therapy use, defined as ≥4 weeks of treatment with drugs such as corticosteroids, disease-modifying antirheumatic agents, and calcineurin inhibitors. The study protocol was approved by the institutional review board of Seoul National University Hospital (No. 2401-052-1500), and the requirement for informed consent was waived because of the retrospective nature of the study. This cohort was included in our previous analyses [8, 9].

Patient characteristics were summarized as counts and proportions for categorical variables and as medians with interquartile ranges (IQRs) for continuous variables. Categorical variables were compared using Pearson's chi-squared test with Yates’ continuity correction, and nonnormally distributed continuous variables were compared using the Kruskal-Wallis test. Culture conversion was defined as at least 3 consecutive negative mycobacterial cultures from sputum samples collected at least 4 weeks apart [10], with the date of the first negative culture considered the conversion date. The hazard ratio (HR) for each drug regarding time to culture conversion (TCC) was analyzed using a time-varying Cox proportional hazards regression model, accounting for antibiotic use duration before the desired event. In the multivariable model, adjustments were made for age, sex, other drugs administered, and cavity presence, treated as a time-varying variable to account for its potential obliteration over time [11]. Statistical analyses were conducted using SAS software (SAS Institute Inc., Cary, NC, USA), with 2-tailed significance levels <0.05 considered statistically significant.

## RESULTS

In total, 534 patients with MAC-PD received macrolide/ethambutol-based regimens. The median age was 64 years (IQR, 57–72), with 67.0% being females. Among the macrolides, azithromycin (66.4%) was used more frequently than clarithromycin (33.6%). Moreover, rifampicin, clofazimine, and aminoglycosides were administered to 378 (70.8%), 102 (19.1%), and 69 (12.9%) patients, respectively (Table 1).

**Table 1.** Baseline and Treatment Characteristics of Patients According to Culture Conversion

Clinical Characteristics	Total (N = 534)	Culture Converted (N = 387)	Culture Nonconverted (N = 147)	*P* Value
Age, y, median (IQR)	64 (57–72)	63 (56–70)	69 (60–74)	<.001
Female, N (%)	358 (67.0)	277 (71.6)	81 (55.1)	<.001
Body mass index, kg/m^2^, median (IQR) (n = 470)	20.8 (19.1–22.6)	20.9 (19.2–22.6)	20.6 (18.7–22.4)	.280
Presence of cavity, N (%) (n = 513)	230 (44.8)	153 (40.9)	77 (55.4)	.005
Macrolide resistance, N (%) (n = 372)	11 (3.0)	2 (0.8)	9 (7.8)	.001
Immunosuppressive therapy use, N (%) (n = 520)	38 (7.3)	30 (8.0)	8 (5.5)	.431
Drugs used, N (%)	…	…	…	
Ethambutol^a^	534 (100.0)	387 (100.0)	147 (100.0)	>.999
Rifampicin	378 (70.8)	284 (73.4)	94 (63.9)	.042
Azithromycin	359 (66.4)	245 (62.3)	113 (76.9)	.002
Clarithromycin	175 (33.6)	142 (37.7)	34 (23.0)	<.001
Clofazimine	102 (19.1)	47 (12.1)	55 (37.4)	<.001
Aminoglycosides	69 (12.9)	34 (8.8)	35 (23.8)	<.001
≥4 drugs used, N (%)	76 (14.2)	32 (8.3)	44 (29.9)	<.001
Treatment duration, d, median (IQR)	526 (358–673)	540 (393–680)	421 (171–644)	<.001

IQR, interquartile range.

^a^All patients included in this study received ethambutol.

Over a median treatment duration of 17.3 months (IQR, 11.8–22.1), 387 patients (72.5%) achieved culture conversion, with a median TCC of 54 days (IQR, 29–133). Patients who achieved culture conversion were younger (*P* < .001) and more often female (*P* < .001). Cavitation was more prevalent among patients who did not achieve culture conversion (55.4% vs 40.9%, *P* = .005). Notably, rifampicin use was higher among patients who achieved culture conversion (73.4% vs 63.9%, *P* = .042), whereas azithromycin, clofazimine, and aminoglycoside use was more common among patients who did not achieve conversion (Table 1).

Time-varying Cox proportional hazards analysis revealed a significant association between rifampicin use and TCC (Table 2). Rifampicin administration was associated with a significantly reduced culture conversion rate, with each additional month of administration yielding an adjusted HR of 0.959 (95% confidence interval [CI], .924–.995; *P* = .027). Conversely, clofazimine (adjusted HR, 1.022; 95% CI, .978–1.069; *P* = .331) and aminoglycosides (adjusted HR, 0.991; 95% CI, .932–1.053; *P* = .774) were not significantly associated with TCC.

**Table 2.** Hazard Ratio for Time to Culture Conversion for Each Drug

Medication	HR	95% CI	*P* Value	Adjusted HR^a^	95% CI	*P* Value
Azithromycin	0.984	.955–1.015	.319	1.005	.972–1.040	.764
Rifampicin	0.997	.964–1.032	.884	0.959	.924–0.995	.027
Clofazimine	0.993	.952–1.036	.744	1.022	.978–1.069	.331
Aminoglycosides	0.976	.915–1.041	.462	0.991	.932–1.053	.774

CI, confidence interval; HR, hazard ratio for each 1-m increase in treatment duration.

^a^Adjusting for age, sex, cavity presence, and concurrently administered drugs.

## DISCUSSION

In this study, we analyzed the effect of individual drug use on time to negative culture conversion in MAC-PD, adjusting for age, sex, cavity presence, and concurrent medications. Our primary finding was that prolonged rifampicin use was associated with a reduced culture conversion rate, whereas clofazimine and aminoglycosides did not significantly affect the culture conversion rate.

MAC-PD requires extended treatment, and our previous study demonstrated a significant association between long-term treatment for ≥18 months and improved outcomes [8]. However, the contribution of individual drugs during this prolonged treatment period remains unclear because of the timing of drug administration and varying durations of drug exposure. Evaluating drug effectiveness solely based on its administration has inherent limitations, particularly when these factors are highly variable [3]. Additionally, drugs introduced later during treatment increase susceptibility to immortal person-time biases [3]. To address this, we quantified individual drug exposure duration and analyzed its effects using a time-varying model. Our results revealed that rifampicin use was inversely correlated with TCC.

At our institution, macrolides and ethambutol are prescribed to all patients with MAC-PD unless contraindicated, whereas rifampicin is prescribed at the physician's discretion. The regimen of patients with more extensive and severe MAC-PD, characterized by cavitary disease, typically includes aminoglycosides, clofazimine, or both [1]. The effect of these drugs on TCC may have been underestimated because of unmeasured, hidden disease severity that could not be fully adjusted for. In contrast, rifampicin was likely administered to less severe cases, as reflected in its higher use among patients who achieved culture conversion. Nevertheless, rifampicin's significant negative association with TCC, after adjusting for clinical characteristics and other concurrent medications, suggests potential evidence of its detrimental impact. The adjusted HR of 0.959 indicates that each additional month of rifampicin use is associated with a 4.1% reduction in the hazard of culture conversion. Over 6 months, this effect would accumulate (0.959^6^ = 0.78), suggesting a slower rate of culture conversion with prolonged rifampicin use.

Rifampicin is recommended as part of the first-line 3-drug regimen with macrolides and ethambutol in international treatment guidelines [1], based on reports suggesting its role in reducing macrolide resistance development and improving survival in patients with MAC-PD [12–14]. However, its use remains controversial because of several factors, including its relatively weak bactericidal activity against MAC [15], its function as a CYP3A4 inducer that may lower macrolide serum concentrations [16], the lack of significant outcome differences according to rifampicin MIC [17], and the failure of recent randomized controlled trials, preclinical, and cohort studies to demonstrate additional benefits when added to macrolide-ethambutol therapy [18–20]. The findings of the present study indicate that rifampicin use is associated with a lower likelihood of favorable treatment outcomes, suggesting the need for further evaluation of its role in MAC-PD treatment.

This study has several limitations. Although treatment regimens were based on international guidelines considering disease severity, patient adherence, and adverse drug reactions, potential bias may have been introduced because of physician discretion. Additionally, we were unable to fully adjust for factors such as drug susceptibility and body mass index due to the higher proportion of missing values [21]. We also could not adjust for unmeasured confounders such as drug dosage variations and underlying disease severity affecting time to culture clearance. Randomized clinical trials, including the ongoing MAC2v3 trial (NCT03672630)[22], are needed to validate our findings.

In conclusion, rifampicin was associated with a reduced TCC rate in MAC-PD. This finding raises the possibility that future treatment strategies for MAC-PD may benefit from evaluating regimens that do not include rifampicin.

## Notes

***Acknowledgments***. None.

***Financial support***. None.
